# Characterization of bronchiectasis in lung cancer using German claims data

**DOI:** 10.1038/s41598-025-34656-2

**Published:** 2026-01-09

**Authors:** Jeremias Götschke, Julia Walter, Kathrin Kahnert, Melanie Götschke, Theodore S. Kapellos, Diego Kauffmann-Guerrero, Jürgen Behr, Amanda Tufman, Pontus Mertsch

**Affiliations:** 1 Department of Medicine V, LMU University Hospital, Comprehensive Pneumology Center (CPC-M), Member of the German Center for Lung Research (DZL), Munich, Germany; 2 Institute of Lung Health and Immunity (LHI), Comprehensive Pneumology Center (CPC-M), Member of the German Center for Lung Research (DZL), Helmholtz Munich, Munich, Germany; 3 Institute of Experimental Pneumology, University Hospital Munich, LMU Munich, Munich, Germany

**Keywords:** Bronchiectasis, Lung cancer, Comorbidity, Survival analysis, Healthcare costs, Pneumonia, Claims data analysis, Lung cancer, Epidemiology

## Abstract

Bronchiectasis is a chronic pulmonary disease characterized by pathological bronchial enlargement due to various underlying etiologies, leading to symptoms such as chronic cough, sputum production, and recurrent respiratory infections. However, the impact of comorbid bronchiectasis on the clinical course of lung cancer remains unclear. This study aims to evaluate the role of bronchiectasis as a comorbidity and complication in patients with lung cancer, specifically in relation to survival, the incidence of pneumonia and healthcare costs. In this retrospective analysis, we utilized claims data from 36,272 German lung cancer patients. Patients with and without comorbid bronchiectasis were matched using propensity score matching (1:1). The minimum follow-up period for all patients was three years. Survival differences were assessed using Kaplan-Meier curves and the Log-Rank test. The occurrence of pneumonia was analyzed using absolute and relative frequencies and compared with a McNemar test for paired samples. To compare costs between the matched groups, we used means with standard deviation and paired t-tests. A total of 572 patients (1.6%) had bronchiectasis, of these, 63.6% were diagnosed prior to lung cancer (PRE bronchiectasis), while 36.4% received the diagnosis thereafter (POST bronchiectasis). No significant difference in survival was observed in the PRE cohort, while the risk for death was significantly higher in bronchiectasis patients in the POST cohort compared to controls (HR = 2.22; 95% CI 1.71–2.87; *p* < 0.001). Among patients undergoing systemic therapy or radiotherapy, patients with bronchiectasis developed pneumonia more often during follow-up, compared patients without bronchiectasis (PRE: 62.4% vs. 45.3%, *p* < 0.001; POST: 64.9% vs. 44.2%, *p* < 0.001). Total costs, as well as expenses for inpatient hospital treatment, doctor visits, and medications, were significantly higher in patients with bronchiectasis. However, after adjusting for survival time, these differences were no longer significant. Pre-existing bronchiectasis does not appear to adversely affect survival in patients with lung cancer. In contrast, incident bronchiectasis developing during follow-up is associated with significantly worse outcomes. Clinically, patients with bronchiectasis should be monitored closely for infectious complications, particularly pneumonia.

**Supplementary Information:**

The online version contains supplementary material available at 10.1038/s41598-025-34656-2.

## Introduction

Lung cancer is the leading cause of cancer-related deaths worldwide^1^. Pulmonary infections are a clinically highly relevant complication during lung cancer treatment^2,3^. Bronchiectasis, a chronic airway disease, has shown an increasing incidence over the last decade and is recognized as a comorbidity in lung cancer^4,5^. Bronchiectasis is defined by pathologic bronchial enlargement and pulmonary symptoms such as chronic cough, phlegm production and often recurrent pulmonary exacerbations^6^. Pneumonia is a frequent and serious complication of lung cancer therapy, with a potentially higher incidence in patients with concurrent bronchiectasis^7^. Despite advances in targeted therapies and immunotherapy for lung cancer, immunosuppressive chemotherapeutic agents remain widely used, further increasing the risk of infections, particularly during neutropenia^8^.

In both bronchiectasis and lung cancer, infections caused by gram-negative bacteria, such as *Pseudomonas aeruginosa*, pose a major concern^9–11^. Patients with bronchiectasis are more susceptible to chronic bacterial infections, including those caused by gram-negative bacteria, making them vulnerable to respiratory complications^12^. Treating these infections often necessitates intensified therapy and incurs higher treatment costs^4,13^. Moreover, patients with lung cancer and pre-existing chronic lung diseases undergoing chemotherapy face an elevated risk of pneumonia and potentially higher mortality rates^14,15^.

Although bronchiectasis is associated with an increased risk of lung cancer, little is known about the relationship between these conditions and the impact of bronchiectasis on the disease course^5^. Given the intricate interplay between bronchiectasis, lung cancer, and infections, a comprehensive understanding of the implications of bronchiectasis in lung cancer patients is essential. This observational cohort study aims to explore the prevalence of pre-existing bronchiectasis in patients with lung cancer; assess its effects on lung cancer treatment, survival, and the occurrence of pneumonia; evaluate the associated healthcare expenditures; and examine newly developed bronchiectasis as a possible treatment-related complication. By analyzing anonymized health insurance claims from a well-defined cohort of patients with a first diagnosis of lung cancer, this study aims to provide insights into the relevance of bronchiectasis in influencing the course of lung cancer treatment and its associated outcomes. This research therefore has the potential to improve tailored treatment strategies, enhance patient care, and optimize health outcomes for individuals with both bronchiectasis and lung cancer.

## Methods

### Data set and sample selection

From all anonymized health insurance claims data available for 2015 and 2016, we identified and included 36,727 patients with an incident (newly diagnosed) lung cancer, as provided by the Scientific Institute of the AOK health insurance trust (WIdO), which covers approximately 30% of the German population^16^.

The study was conducted in accordance with all relevant guidelines and regulations, specifically adhering to the Good Practice of Secondary Data Analysis guidelines^17^. As the analysis involved only fully anonymized secondary data, ethical approval and informed consent were not required. A waiver of ethical approval and informed consent therefore applies.

Basic data contained year of birth, sex, and regional type of residence (major city, urban, rural, remote rural). Additionally, we used claims for hospitalization, outpatient hospital care, outpatient doctor visits and medications for a period of three years after diagnosis. These included German International Classification of Diseases (ICD-10-GM) codes, OPS codes (German Version of the International Classification of Procedures in Medicine), billing codes (GONR) and Anatomical Therapeutic Chemical (ATC) codes.

We identified our study sample according to a three-step process. First, we selected all patients with a diagnosis of lung cancer (ICD-10_GM: C34) in 2015 and 2016. In a second step, to avoid false positives, we only included patients with at least one inpatient principal diagnosis or at least two confirmed outpatient diagnoses in two distinct quarters in the year 2015 or 2016. Third, we excluded all patients with a history of lung cancer or lung metastases in the two years prior to the diagnosis.

### Diagnosis of bronchiectasis

We identified patients with bronchiectasis using ICD-10 GM code J47. Patients with bronchiectasis were further categorized into patients with a documented diagnosis of bronchiectasis pre or post lung cancer diagnosis. As we suspected that some patients may have had undiagnosed bronchiectasis, which was only detected during lung cancer diagnosis, we defined ‘pre lung cancer bronchiectasis’ (PRE bronchiectasis) as a documented ICD code J47 up to 90 days after lung cancer diagnosis. ‘Post lung cancer bronchiectasis’ (POST bronchiectasis) was defined as bronchiectasis documented the first time more than 90 days after the diagnosis of lung cancer.

### Patient characteristics

Comorbidities were measured using the Charlson comorbidity index (modified by excluding lung cancer), which was calculated based on all inpatient and confirmed outpatient ICD diagnoses in the two years preceding the initial lung cancer diagnosis. As tumor stage is not available in German claims data, we approximated disease stage using ICD codes for secondary malignancies recorded within three months of the initial lung cancer diagnosis. Patients were categorized as follows: no lymph node involvement or metastases (N0, M0), lymph node involvement without distant metastases (N1, N2, N3), lung metastases (Ma), and distant metastases (Mb, Mc). Among patients classified as N0, M0, we further differentiated between those with documented diagnostic imaging (e.g., CT, PET-CT) within three months of diagnosis and those without such diagnostic evaluation. To measure hospital size, we identified the hospital where each patient received the majority of their lung cancer care. Based on this information, we categorized hospitals by the number of patients receiving treatment: fewer than 50, 50 to 99, 100 to 199, 200 to 299, 300 to 399, and more than 400 patients.

### Outcome parameters

We compared the following outcomes between patients with and without bronchiectasis: therapy, occurrence of pneumonia, survival, hospitalizations and costs.

We used OPS codes, GONR codes, and ATC codes to identify which treatments patients received during the initial phase (90 days after diagnosis) and over the course of the disease. We identified lung cancer resections (atypical and anatomic), receipt of radiotherapy, and receipt of systemic therapy. Therapy was assessed for the whole duration of follow-up as well as for the first three months after diagnosis.

We assessed overall survival using the date of death or the date of last follow-up, whichever occurred first. All patients had a minimum follow-up duration of three years after diagnosis, with a maximum observation period of five years. Survival time was censored at the date of the last recorded follow-up. To account for immortal-time bias, as the development of bronchiectasis in the POST cohort was time-dependent, we applied a time-dependent Cox model to assess survival during follow-up.

To assess the occurrence of pneumonia we used ICD codes relating to pneumonia (J11 – J18) documented during the course of the disease, and calculated proportions of patients with pneumonia in the subgroup of patients who received radio- or systemic therapy. Costs (expenditures for the insurance company) were calculated as the total sum of all expenditures in the three years after diagnosis for inpatient and outpatient hospitalizations, outpatient physician visits, drugs and rehabilitation, and remedies and adjuvants. Lung cancer-related costs were considered as a subset of these total costs and were identified using the ICD code C34 for hospital visits (principal diagnosis), physician visits, rehabilitation, and remedies and adjuvants. For medications, we applied ATC codes specific to lung cancer-related drugs.

Additionally, we calculated the number of hospitalizations, and the number of hospital days for each patient, as well as the lung cancer-related hospitalizations and hospital days (identified by principal diagnosis of C34). Because these outcomes depend on the length of survival, we standardized them by dividing the raw counts by the number of months each patient survived and multiplying the result by 12. This yielded annualized estimates, allowing comparisons independent of individual survival time.

### Statistical analysis

Patient characteristics were presented as absolute and relative frequencies, and means with standard deviation, and compared using standardized differences. We used propensity score matching with nearest neighbor criteria to separately match patients with PRE and POST bronchiectasis to controls without bronchiectasis using baseline characteristics with a standardized difference (stdiff) of greater than 0.1. To assess the time until bronchiectasis development for the POST bronchiectasis group, we used cumulative incidence curves.

We visually inspected three-year survival using Kaplan Meier curves and compared survival between the three groups using log-rank test. Therapies and the occurrence of pneumonia were reported as absolute and relative frequencies and compared using McNemar test for matched samples. Costs and hospitalization data were reported as means with standard deviation and compared paired t-test.

A significance threshold of 0.05 was applied for all analyses. All analyses were performed using R Studio with R version 4.0; tables and figures were created in Microsoft Excel and R Studio^18^.

## Results

### Patient characteristics

After inspecting standardized differences between patients with and without bronchiectasis we used sex, metastases status, regional residence type, hospital size, congestive heart disease, peripheral vascular disease, and diabetes with complications in the propensity matching of the PRE bronchiectasis group. In the POST bronchiectasis cohort we additionally used age, and only the comorbidity hemi- and paraplegia. This resulted in a cohort of 364 patients and controls in the PRE group and 208 patients and controls in the POST group. Patient characteristics after propensity score matching stratified by bronchiectasis are displayed in Table 1. Characteristics before matching are displayed in supplemental Table S1.

**Table 1:** Patient characteristics after propensity score matching, stratified by diagnosis of bronchiectasis.

	PRE bronchiectasis (*n* = 364)		PRE controls (*n* = 364)		stdiff	POST bronchiectasis (*n* = 208)		POST controls (*n* = 208)		stdiff
	mean	sd	mean	sd		mean	sd	mean	sd	
Age	70.0	10.3	69.7	10.1	0.03	68.2	9.4	69.0	9.1	0.09
CCI score	3.4	2.19	3.1	2.24	0.14	2.8	2.08	2.6	2.09	0.06
	n	%	n	%	stdiff	n	%	n	%	stdiff
Sex										
Male	258	70.9%	258	70.9%		155	74.5%	163	78.4%	
Female	106	29.1%	106	29.1%	0.00	53	25.5%	45	21.6%	0.09
Metastases status at diagnosis										
N0,M0 & diagnostic	140	38.5%	141	38.7%		66	31.7%	68	32.7%	
N0,M0 & no diagnostic	27	7.4%	26	7.1%		28	13.5%	28	13.5%	
N1,N2,N3	57	15.7%	57	15.7%		45	21.6%	42	20.2%	
Ma	34	9.3%	34	9.3%		23	11.1%	20	9.6%	
Mb, Mc	106	29.1%	106	29.1%	0.01	46	22.1%	50	24.0%	0.07
Regional type of residence										
Major city	116	31.9%	115	31.6%		63	30.3%	67	32.2%	
Urban	119	32.7%	119	32.7%		80	38.5%	80	38.5%	
Rural	70	19.2%	71	19.5%		40	19.2%	40	19.2%	
Remote rural	59	16.2%	59	16.2%	0.01	25	12.0%	21	10.1%	0.07
Unknown										
Hospital size (# of lung cancer patients)										
Fewer than 50	40	11.0%	40	11.0%		14	6.7%	10	4.8%	
50–99	51	14.0%	51	14.0%		26	12.5%	26	12.5%	
100–199	109	29.9%	110	30.2%		66	31.7%	68	32.7%	
200–299	71	19.5%	71	19.5%		38	18.3%	41	19.7%	
300–399	55	15.1%	54	14.8%		42	20.2%	38	18.3%	
400 and more	38	10.4%	38	10.4%	0.01	22	10.6%	25	12.0%	0.11
Unknown										
Comorbidities										
Myocardial infarction	47	12.9%	53	14.6%	0.05	17	8.2%	26	12.5%	0.14
Congestive heart disease	118	32.4%	118	32.4%	0.00	48	23.1%	43	20.7%	0.06
Peripheral vascular disease	143	39.3%	144	39.6%	0.01	75	36.1%	82	39.4%	0.07
Cerebrovascular disease	81	22.3%	79	21.7%	0.01	40	19.2%	31	14.9%	0.12
dementia	24	6.6%	21	5.8%	0.03	13	6.3%	12	5.8%	0.02
Chronic pulmonary disease	316	86.8%	212	58.2%	0.68	140	67.3%	117	56.3%	0.23
Rheumatoid arthritis	15	4.1%	21	5.8%	0.08	11	5.3%	9	4.3%	0.05
Peptic ulcer disease	18	4.9%	18	4.9%	0.00	10	4.8%	7	3.4%	0.07
Mild liver disease	74	20.3%	64	17.6%	0.07	41	19.7%	33	15.9%	0.10
Diabetes without complication	119	32.7%	122	33.5%	0.02	59	28.4%	68	32.7%	0.09
Diabetes with complication	79	21.7%	79	21.7%	0.00	33	15.9%	33	15.9%	0.00
Hemi- & paraplegia	18	4.9%	18	4.9%	0.00	2	1.0%	2	1.0%	0.00
renal disease	77	21.2%	75	20.6%	0.01	36	17.3%	29	13.9%	0.09
Prior cancer	83	22.8%	63	17.3%	0.14	34	16.3%	47	22.6%	0.16
Mild to severe liver disease	5	1.4%	6	1.6%	0.02	3	1.4%	1	0.5%	0.10
Prior metastatic cancer	35	9.6%	26	7.1%	0.09	16	7.7%	8	3.8%	0.17
HIV/AIDS	0	0.0%	1	0.3%	0.07	0	0.0%	0	0.0%	0.00

Comparison of patient characteristics after propensity score matching of patients with and without bronchiectasis in the context of lung cancer, using standardized differences. *PRE bronchiectasis*: bronchiectasis diagnosed at the time of lung cancer diagnosis; *PRE controls*: no bronchiectasis diagnosed at the time of lung cancer diagnosis; *POST bronchiectasis*: bronchiectasis diagnosed after lung cancer diagnosis; *POST controls*: no bronchiectasis diagnosed after lung cancer diagnosis. Age and sex are reported as mean ± standard deviation; categorical variables are presented as absolute and relative frequencies.

Abbreviations: Charlson Comorbidity Index, CCI; standardized difference, stdiff; standard deviation, SD; Human Immunodeficiency Virus, HIV; Acquired Immune Deficiency Syndrome, AIDS.

### Timing of bronchiectasis diagnosis

**Fig. 1:**
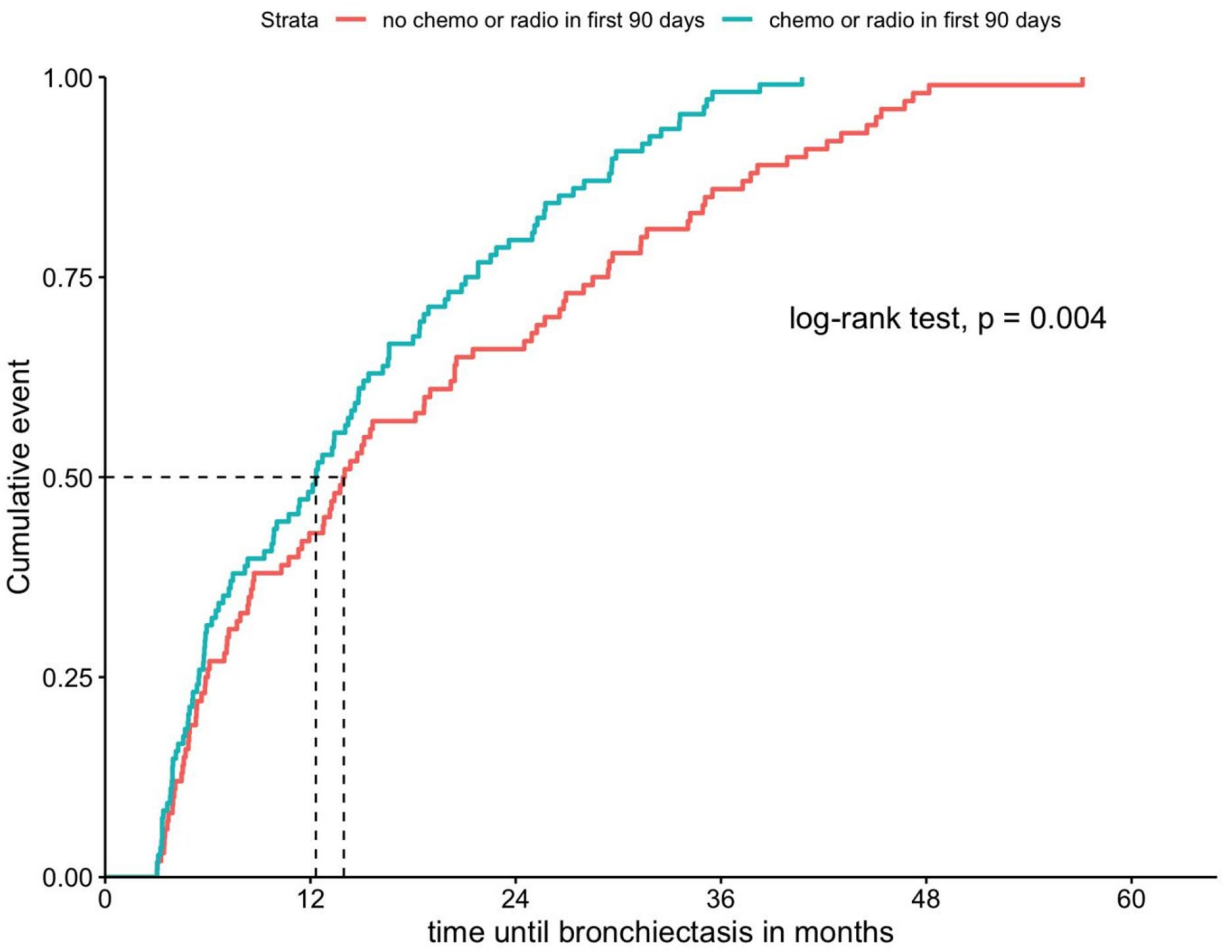
Cumulative incidence of POST bronchiectasis diagnosis, stratified by initial therapy. *POST bronchiectasis*: first ICD coding of bronchiectasis (J47) occurring at least 90 days after initial lung cancer diagnosis.

In total, 1.6% (*n* = 572) of all patients with lung cancer also had a diagnosis of bronchiectasis. Of these 364 (63.6%) were in the PRE group and 208 (36.4%) in the POST group. The median time to bronchiectasis of patients with POST bronchiectasis was 13.2 months. When stratified by receipt of systemic- or radiotherapy in the first 90 days after lung cancer diagnosis, the median time to bronchiectasis was 12.3 months for those who received therapy, and 13.9 months for those who did not (*p* = 0.004, Log-rank test). Cumulative incidence curve for time to bronchiectasis within the POST is displayed in Fig. 1.

### Therapy

**Fig. 2:**
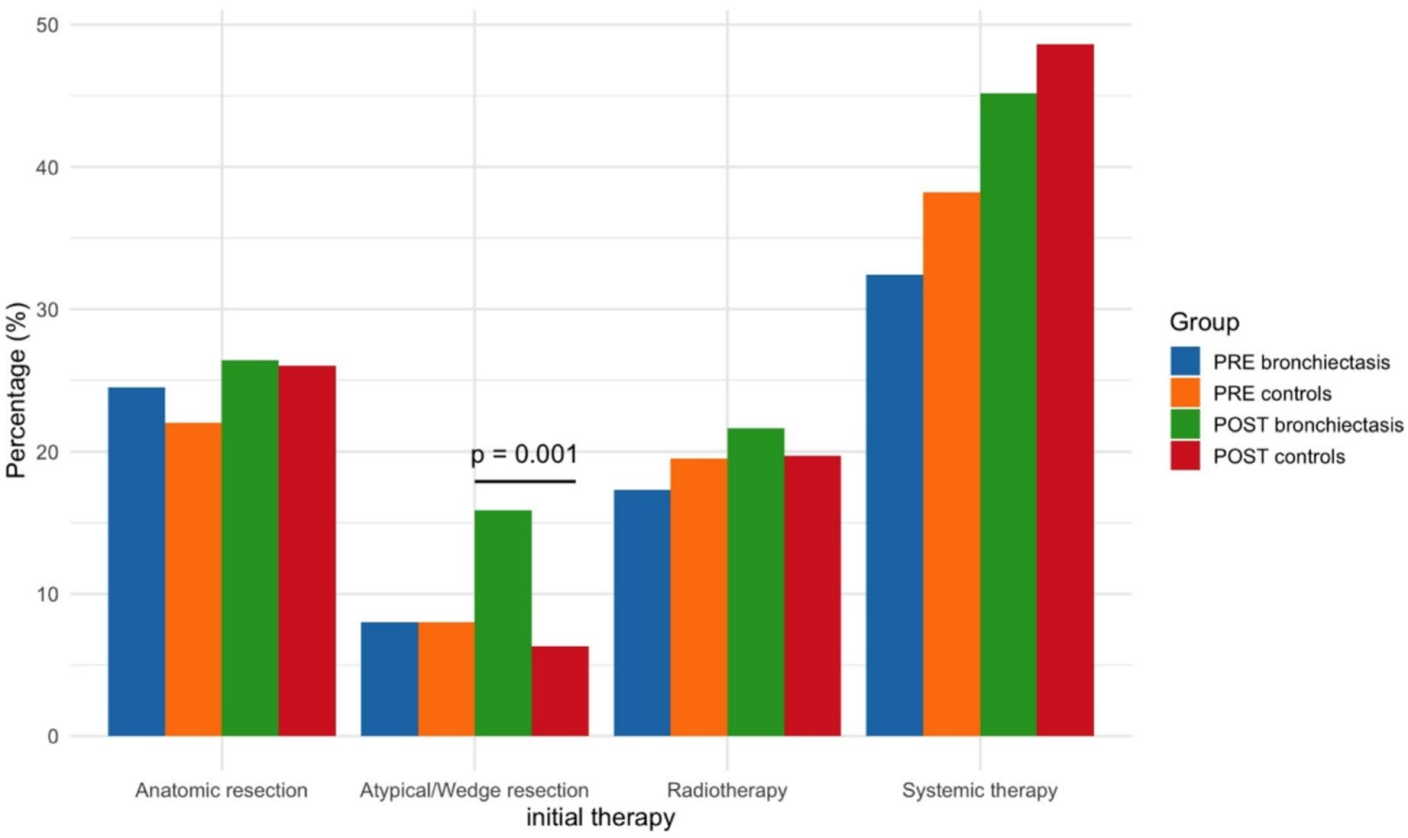
Initial lung cancer therapy stratified by bronchiectasis status. The only statistically significant difference was atypical/wedge resections between patients with bronchiectasis and matched controls within the post-bronchiectasis cohort. Abbreviations: *PRE bronchiectasis*: bronchiectasis diagnosed at the time of lung cancer diagnosis; *PRE controls*: no bronchiectasis diagnosed at the time of lung cancer diagnosis; *POST bronchiectasis*: bronchiectasis diagnosed after lung cancer diagnosis; *POST controls*: no bronchiectasis diagnosed after lung cancer diagnosis.

Within the first three months following lung cancer diagnosis, 32.4% of patients with PRE bronchiectasis received systemic therapy compared to 38.2% of matched controls (*p* = 0.10). In the POST cohort, the corresponding proportions were 45.2% and 48.6%, respectively (*p* = 0.51). In percentage terms, the proportion of systemic therapy was lower in the PRE group than in the POST group. Among POST patients, 15.9% had a wedge resection in the first three months compared to 6.3% of controls (*p* = 0.001). For further details please refer to Fig. 2.

**Fig. 3:**
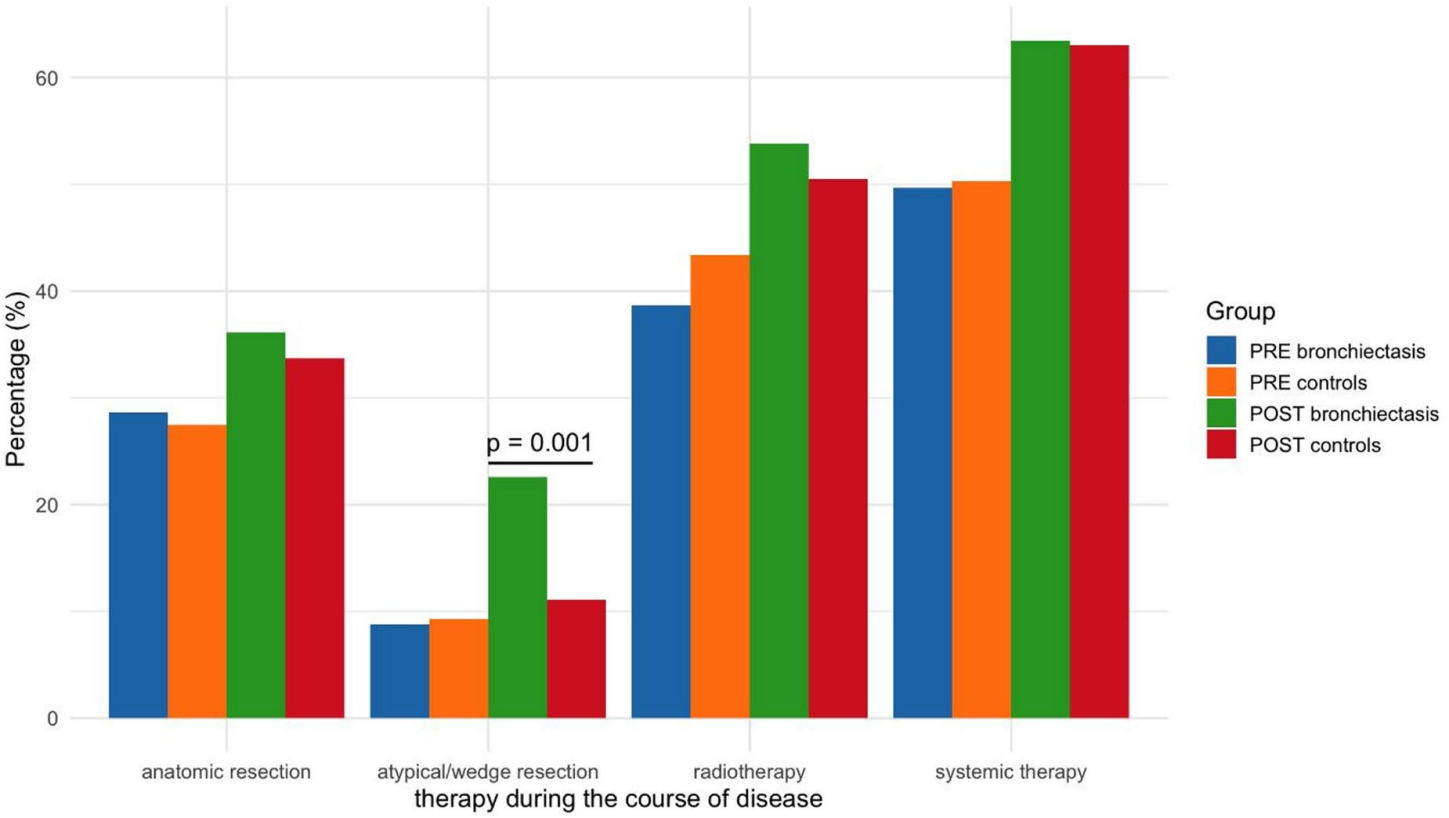
Lung cancer therapy during the course of disease stratified by bronchiectasis status. The only statistically significant difference was atypical/wedge resections between patients with bronchiectasis and matched controls within the post-bronchiectasis cohort. Abbreviations: *PRE bronchiectasis*: bronchiectasis diagnosed at the time of lung cancer diagnosis; *PRE controls*: no bronchiectasis diagnosed at the time of lung cancer diagnosis; *POST bronchiectasis*: bronchiectasis diagnosed after lung cancer diagnosis; *POST controls*: no bronchiectasis diagnosed after lung cancer diagnosis.

Overall proportions of several therapy modalities were higher in the POST group than in the PRE cohort. Statistical comparisons confirmed that wedge resections, radiotherapy and systemic therapy occurred significantly (all *p* < 0.05) more often in the POST bronchiectasis group, while anatomic resections did not differ significantly between periods. Figure 3 displays the therapy over the course of disease in detail. Supplement Table S1 shows the comparisons for PRE and POST overall and initial treatment for bronchiectasis and controls in detail.

### Survival

**Fig. 4:**
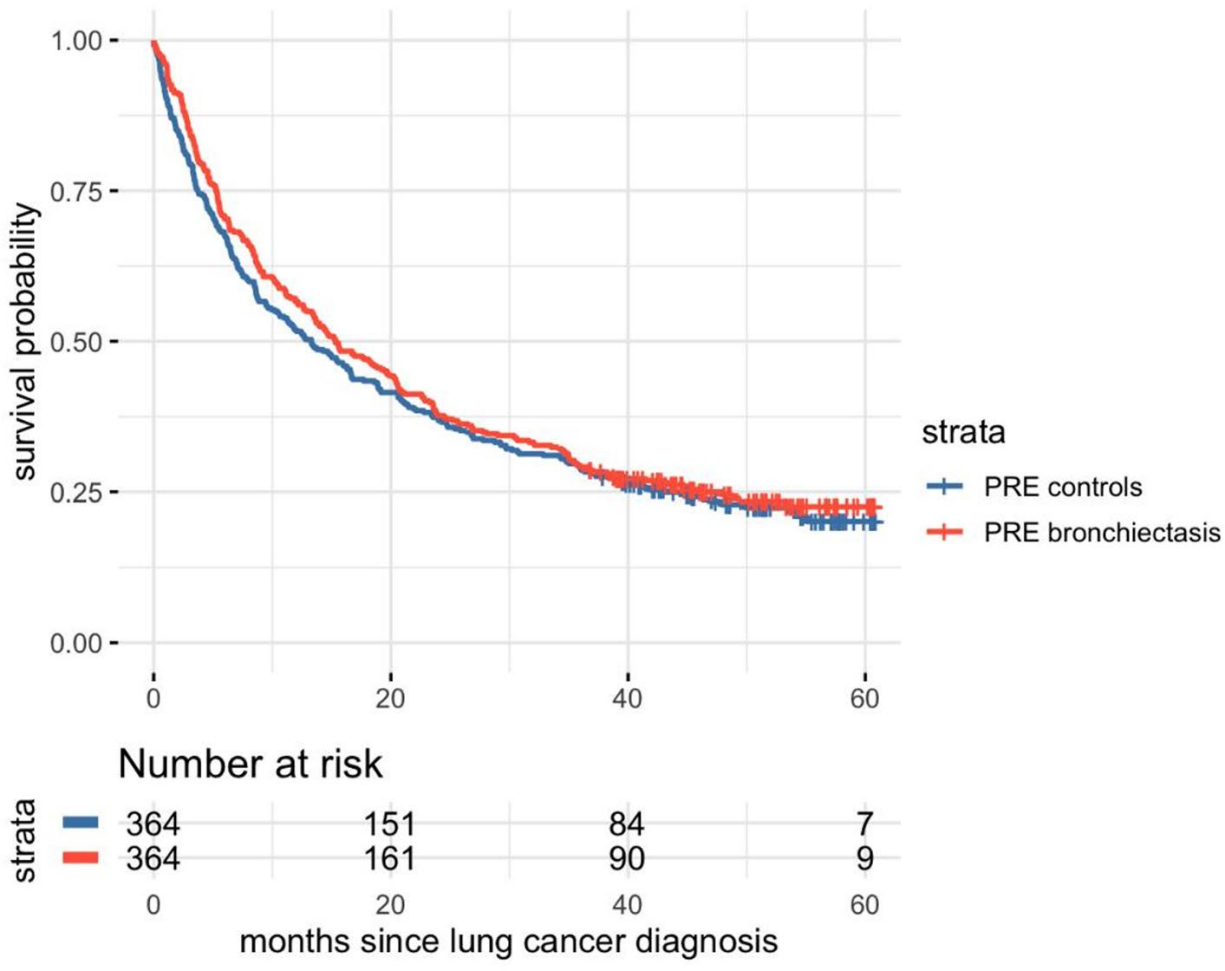
Survival compared to matched controls in the PRE cohort. Survival analysis of the PRE cohort revealed no significant difference between PRE bronchiectasis and control (*p* = 0.36). Abbreviations: *PRE bronchiectasis*: bronchiectasis diagnosed at the time of lung cancer diagnosis; *PRE control*s: no bronchiectasis diagnosed at the time of lung cancer diagnosis.

Survival differed not significantly between patients with PRE bronchiectasis and matched controls (*p* = 0.36). Median survival time for patients with PRE bronchiectasis was 15.3 months compared to 13.3 months for controls (Fig. 4). Using a time-dependent Cox proportional hazards model to account for varying times of bronchiectasis onset in the POST cohort, incident bronchiectasis was associated with a significantly increased risk of death (HR 2.22, 95% CI 1.71–2.87, *p* < 0.001) (Figure S2).

### Pneumonia and nontuberculous mycobacteria infection

The proportion of patients developing pneumonia over the course of disease was significantly higher in all patients with bronchiectasis compared to controls (PRE group: 62.4% vs. 45.3%, *p* < 0.001; POST group: 64.9% vs. 44.2%, *p* < 0.001). The mean number of pneumonias per patient was also significantly higher in all patients with bronchiectasis compared to controls, and in both, the PRE cohort (1.4 vs. 1.0, *p* = 0.01) and the POST cohort (1.9 vs. 0.9, *p* < 0.001). To further explore the temporal relationship between pneumonia and POST bronchiectasis, we analyzed the timing of pneumonia events relative to the diagnosis of bronchiectasis in the POST cohort (Figure S1). Among 208 patients with POST bronchiectasis, 135 (64.9%) had documented pneumonia, with pneumonia occurring significantly more often before than after bronchiectasis onset (103 vs. 32 patients; *p* < 0.001).

Two patients in the analysis had a nontuberculous mycobacteria infection after their lung cancer diagnosis; both were patients with bronchiectasis, one in the PRE and one in the POST bronchiectasis group.

### Expenditures

Expenditures did not differ significantly between patients with bronchiectasis and controls in the PRE group. All-cause and lung cancer specific expenditures were significantly higher in patients with POST bronchiectasis compared to controls. Additionally, all-cause and lung cancer-specific domain specific expenditures for hospitalizations, outpatient doctor visits and rehabilitation were significantly higher in patients with POST bronchiectasis (Table 2). When adjusted for survival time, expenditures did not differ significantly anymore. Expenditures overall and in all domains as well as lung cancer-related expenditures are displayed in Table 3.

**Table 2:** Unadjusted 3-year expenditures stratified by bronchiectasis diagnosis.

	PRE bronchiectasis (*n* = 364)		PRE controls (*n* = 364)		*p*-value	POST bronchiectasis (*n* = 208)		POST controls (*n* = 208)		*p*-value
	Mean	sd	Mean	sd		Mean	sd	Mean	sd	
All costs										
Total costs	47,655 €	43,149 €	46,494 €	44,519 €	0.72	74,983 €	64,130 €	53,581 €	46,467 €	0.0001
Hospital costs	29,370 €	25,023 €	27,350 €	22,787 €	0.26	39,426 €	30,319 €	28,260 €	18,217 €	< 0.0001
Outpatient physician costs	4,349 €	4,620 €	4,630 €	5,029 €	0.43	7,489 €	5,546 €	5,814 €	6,909 €	0.01
Costs for drugs	13,460 €	30,820 €	14,006 €	32,591 €	0.82	27,469 €	53,121 €	19,086 €	38,083 €	0.07
Costs for rehabilitation	314 €	1,668 €	273 €	1,375 €	0.72	396 €	1,288 €	171 €	883 €	0.04
Costs for remedies and adjuvants	160 €	506 €	234 €	724 €	0.11	203 €	591 €	249 €	811 €	0.51
Lung cancer related costs										
Total costs	23,676 €	26,162 €	22,744 €	20,725 €	0.59	34,269 €	31,435 €	26,470 €	21,301 €	0.003
Hospital costs	19,481 €	19,040 €	19,253 €	18,182 €	0.87	26,650 €	23,831 €	20,154 €	14,654 €	0.001
Outpatient physician costs	244 €	697 €	293 €	823 €	0.38	566 €	1,051 €	356 €	933 €	0.03
Costs for drugs	3,910 €	16,507 €	3,141 €	9,426 €	0.44	6,884 €	18,104 €	5,941 €	14,791 €	0.56
Costs for rehabilitation	41 €	347 €	57 €	404 €	0.58	169 €	869 €	19 €	270 €	0.02

Health insurance expenditures of patients with lung cancer during the three years following diagnosis, reported as mean ± standard deviation. P-values are derived from paired t-tests for pooled samples.

Abbreviations: *PRE bronchiectasis*: bronchiectasis diagnosed at the time of lung cancer diagnosis; *PRE controls*: no bronchiectasis diagnosed at the time of lung cancer diagnosis; *POST bronchiectasis*: bronchiectasis diagnosed after lung cancer diagnosis; *POST controls*: no bronchiectasis diagnosed after lung cancer diagnosis; sd: standard deviation.

**Table 3:** Expenditures per month adjusted for survival time, stratified by bronchiectasis diagnosis.

	PRE bronchiectasis (*n* = 364)		PRE controls (*n* = 364)		*p*-value	POST bronchiectasis (*n* = 208)		POST controls (*n* = 208)		*p*-value
	Mean	sd	Mean	sd		Mean	sd	Mean	sd	
All costs										
Total costs	4,604 €	6,905 €	5,783 €	11,507 €	0.09	2,968 €	2,866 €	2,967 €	2,293 €	1.00
Hospital costs	3,669 €	6,900 €	4,785 €	11,027 €	0.10	1,793 €	2,435 €	1,870 €	1,844 €	0.72
Outpatient physician costs	283 €	364 €	322 €	522 €	0.25	278 €	266 €	266 €	274 €	0.64
Costs for drugs	618 €	998 €	618 €	987 €	1.00	878 €	1,516 €	814 €	1,299 €	0.65
Costs for rehabilitation	29 €	285 €	56 €	877 €	0.58	11 €	40 €	5 €	33 €	0.09
Costs for remedies and adjuvants	7 €	21 €	8 €	20 €	0.85	7 €	15 €	11 €	27 €	0.07
Lung cancer related costs										
Total costs	3,079 €	6,657 €	4,342 €	10,990 €	0.06	1,539 €	2,043 €	1,727 €	1,664 €	0.30
Hospital costs	2,884 €	6,668 €	4,152 €	11,016 €	0.06	1,272 €	1,951 €	1,401 €	1,511 €	0.45
Outpatient physician costs	5 €	14 €	6 €	18 €	0.40	12 €	24 €	8 €	23 €	0.07
Costs for drugs	190 €	540 €	184 €	501 €	0.88	250 €	581 €	316 €	722 €	0.30
Costs for rehabilitation	2 €	15 €	2 €	11 €	0.90	5 €	28 €	1 €	20 €	0.11

Monthly health insurance expenditures adjusted for survival time in patients with lung cancer.

Abbreviations: *PRE bronchiectasis*: bronchiectasis diagnosed at the time of lung cancer diagnosis; *PRE controls*: no bronchiectasis diagnosed at the time of lung cancer diagnosis; *POST bronchiectasis*: bronchiectasis diagnosed after lung cancer diagnosis; *POST controls*: no bronchiectasis diagnosed after lung cancer diagnosis; sd: standard deviation.

## Discussion

This study investigated the implications of bronchiectasis as a comorbidity and complication in lung cancer patients and its impact on therapy, survival, pneumonia occurrence and healthcare expenditures. It provides valuable insights into the relationship between bronchiectasis and lung cancer, shedding light on potential clinical implications.

The study reported a prevalence of bronchiectasis in 1.6% of all lung cancer patients, with about two thirds of cases diagnosed prior to lung cancer and 36.5% occuring during the course of the disease. Recent studies in Germany report a prevalence of bronchiectasis ranging from 52.5 to 94.8 per 100,000^4^. Our study suggests that the prevalence of bronchiectasis in patients with lung cancer is slightly above the expected prevalence for the general population. This may be explained by smoking status and the increased risk COPD development, which not only increases the risk of lung cancer but is also the third most relevant etiology of bronchiectasis^19,20^.This finding highlights the importance of considering bronchiectasis as a relevant comorbidity in lung cancer patients.

Interestingly, patients with PRE bronchiectasis had a lower proportion of systemic therapy in the first three months after lung cancer diagnosis compared to controls. This finding suggests that the presence of bronchiectasis may influence treatment decisions or might be associated with underlying factors affecting therapeutic choices. It further suggests that clinicians may be more cautious in administering certain treatments to lung cancer patients with pre-existing bronchiectasis due to concerns about potential exacerbation of respiratory symptoms or increased susceptibility to infections.

Conversely, patients with POST lung cancer bronchiectasis had a higher overall treatment burden, including a significantly higher rate of atypical or wedge resections, which may reflect a more aggressive therapeutic approach. Additionally, the greater use of chemotherapy and radiotherapy in this group may contribute to the development of bronchiectasis. Both therapies can impair immune function, increasing susceptibility to respiratory infections, which can trigger chronic inflammation and airway damage, ultimately leading to bronchiectasis^21–24^. Immunosuppression and chemotherapy have also been described in pediatric populations as factors contributing to bronchiectasis, with a mean latency of 34 months (8–333 months)^25^.

In our analysis of the POST cohort, 135 of 208 patients had documented pneumonia, with the majority occurring before the diagnosis of bronchiectasis. This suggests that roughly half of POST bronchiectasis cases may have a post-infectious origin, potentially related to immunosuppression from oncologic therapy. Of the remaining patients, approximately 50% did not have documented pneumonia before the first coding of bronchiectasis, indicating that other factors, such as treatment-related effects or tumor-associated mechanisms, might have contributed to bronchiectasis development in these cases. These findings are broadly consistent with results from bronchiectasis registries, where post-infectious disease is the second most common etiology^26^. From a clinical perspective, the contribution of bronchiectasis to the overall respiratory status of lung cancer patients may often be overshadowed by the severity of the underlying malignancy and by frequent comorbid respiratory conditions with overlapping symptoms. Pneumonia is a serious complication in lung cancer patients, associated with substantial morbidity and mortality^27^. Vaccination with available vaccines, together with early identification and appropriate management of respiratory infections are therefore crucial to reduce the risk of bronchiectasis and improve patient outcomes. Consistent with this, our study demonstrated a significantly higher prevalence of pneumonia among patients with bronchiectasis in both the PRE and POST cohorts, highlighting the complex interplay between lung cancer, its treatment, and subsequent respiratory complications.

Interestingly, patients with PRE bronchiectasis showed no difference in survival compared with their matched controls. In contrast, in the POST cohort, survival after the onset of bronchiectasis was significantly worse in the time-dependent analysis. The underlying reasons for these differences cannot be fully elucidated based on our data, but they are likely multifactorial in origin. One possible explanation might be that patients who developed bronchiectasis during follow-up may have had poorer baseline functional status, which is known to negatively affect survival in lung cancer^14,15,28^. Functional status, such as ECOG (Eastern Cooperative Oncology Group) performance score, is not captured in the claims dataset and is only indirectly approximated by the number of comorbidities, but without information on the severity of individual conditions.

Propensity score matching may still allow for minor residual differences. For example, although the overall comorbidity burden did not differ significantly, patients in the POST bronchiectasis group had a higher prevalence of chronic pulmonary disease. In addition, the TNM stage can only be approximated in the dataset, limiting precise adjustment for tumor burden.

Moreover, the POST group exhibited a higher incidence of pneumonia, a complication associated with increased mortality in lung cancer and indicative of a greater inflammatory burden^29^. Since systemic inflammation is a recognized contributor to poorer outcomes, the elevated pneumonia rates in the POST cohort may partially explain the observed survival differences^30,31^. Collectively, these factors might contribute to the worse prognosis in the POST cohort.

Patients with POST lung cancer bronchiectasis showed higher healthcare expenditures compared to controls, but this difference was no longer significant after adjusting for survival time. This result suggests that the increased expenditures were driven by longer survival rather than direct implications of bronchiectasis on healthcare costs. However, patients with bronchiectasis and lung cancer may require more comprehensive and long-term medical care, including management of chronic respiratory infections, exacerbations, and pulmonary rehabilitation. Moreover, it has been shown that patients with bronchiectasis have an increased healthcare resource utilization^32^.

Our findings emphasize the importance of recognizing bronchiectasis as a comorbidity in lung cancer patients. Identifying and managing bronchiectasis in this population might potentially lead to better respiratory outcomes and reduce the risk of pneumonia. Moreover, the study underscores the need for individualized treatment approaches that consider the presence of bronchiectasis, especially during the diagnosis and planning of lung cancer therapy.

## Limitations

While the study provides valuable insights, several limitations should be acknowledged. The retrospective nature of the analysis using health insurance claims data may have introduced some biases or confounding factors. Due to the coding of the data, it is not possible to differentiate between the descriptive radiological definition of bronchiectasis and clinically relevant bronchiectasis, which is associated with corresponding symptoms. Because the dataset does not capture symptom information, this distinction cannot be made according to current consensus diagnostic criteria^6^. However, the findings of this study suggest that patients with bronchiectasis and lung cancer are more frequently affected by pulmonary infections, a defining feature of clinically relevant bronchiectasis, and thereby distinguishing it from purely radiological abnormalities. In the context of a life-threatening disease such as lung cancer, bronchiectasis may only be coded once it is sufficiently pronounced, increasing the likelihood that the recorded cases represent clinically meaningful disease. In this context it is also possible that the data are subject to observer bias or selective reporting bias. Additionally, the study’s sample size may limit the generalizability of the results.

Performance status was not available in our dataset and could therefore not be included in the matching. Although cohorts were balanced using the Charlson Comorbidity Index, this does not fully account for functional status. As performance status is an independent predictor of survival and treatment tolerance in lung cancer, residual confounding cannot be excluded^28^.

Future research with larger, prospective cohorts could validate and expand the findings.

## Conclusion

This study underscores the clinical relevance of bronchiectasis as a comorbidity and as complication in patients with lung cancer. While pre-existing bronchiectasis does not appear to negatively affect survival, bronchiectasis developing during the course of treatment is associated with significantly worse outcomes. The higher incidence of pneumonia in patients with bronchiectasis highlights the need for timely detection and appropriate management. Clinicians should remain vigilant for bronchiectasis-related complications when making therapy decisions, particularly in patients undergoing systemic therapy or radiotherapy. Future research should aim to further clarify the underlying mechanisms and to optimize care strategies for this vulnerable patient population.

## Supplementary Information

Below is the link to the electronic supplementary material.

Supplementary Material 1

## Data Availability

The authors confirm that the data utilized in this study cannot be made available in the manuscript, the supplemental files, or in a public repository due to German data protection laws (‘Bundesdatenschutzgesetz’, BDSG). Therefore, they are stored on a secure drive in the senior author’s institution to facilitate replication of the results.Generally, access to data of statutory health insurance funds for research purposes is possible only under the conditions defined in German Social Law (SGB V § 287). Requests for data access can be sent as a formal proposal specifying the recipient and purpose of the data transfer to the appropriate data protection agency. Access to the data used in this study can only be provided to external parties under the conditions of the cooperation contract of this research project and after written approval by the sickness fund. For assistance in obtaining access to the data, please contact the corresponding author.
