# Supplementary material for: Characterization of bronchiectasis in lung cancer using German claims data
Source: Sci Rep. 2026 Jan 9;16:1619. doi: 10.1038/s41598-025-34656-2 (PMC12800075; doi:10.1038/s41598-025-34656-2)
Supplement: Supplementary file 1 — Supplementary Material 1 [file 41598_2025_34656_MOESM1_ESM.docx]

**Supplement**


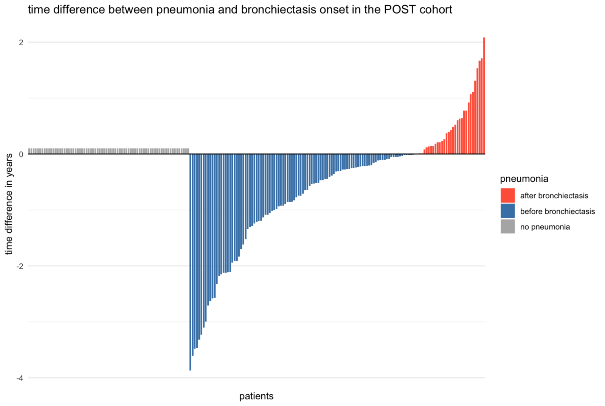
 
Figure S1: Time difference between pneumonie und bronchiectasis onset in the POST cohort.

Each bar represents a patient with documented pneumonia (n = 135). Bars extending downward indicate pneumonia events occurring before the diagnosis of POST, and bars extending upward indicate events occurring after diagnosis. Patients without documented pneumonia (n = 73) are shown on the left with grey bars as placeholders. The plot demonstrates that most pneumonia events precede the onset of POST bronchiectasis, supporting a possible postinfectious etiology in a substantial proportion of cases.


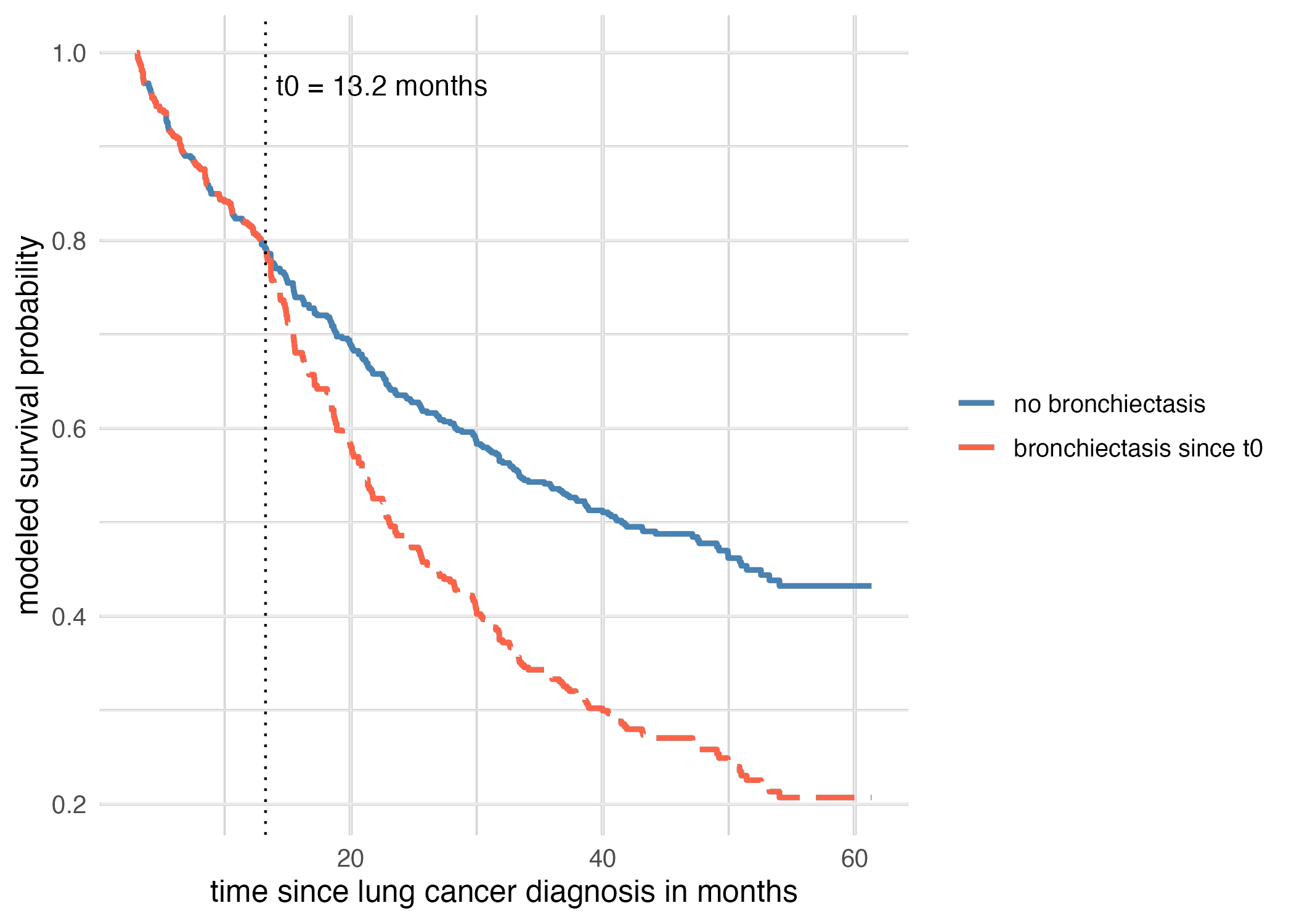
**Figure S2: Time-dependent Cox model for incident bronchiectasis in the POST cohort.**

The two-colored line indicates the median time to first bronchiectasis (t0 = 13.2 months). Before t0, survival is identical for all patients. After t0, the model contrasts patients without bronchiectasis (blue) with a counterfactual scenario where all develop bronchiectasis (red), showing worse survival associated with bronchiectasis (HR = 2.22; 95 % CI 1.71–2.87; p<0.001).

| Treatment overall | | | | | | |
| --- | --- | --- | --- | --- | --- | --- |
|  | Bronchiectasis | | | Controls | | |
|  | PRE (n=364) | POST (n=208) | p | PRE (n=364) | POST (n=208) | p |
| atypical/wedge resection, n(%) | 32 (8.8%) | 47 (22.6%) | <0.001 | 34 (9.3%) | 23 (11.1%) | 0.51 |
| Anatomic resection, n(%) | 104 (28.6%) | 75 (36.1%) | 0.063 | 100 (27.5%) | 70 (33.7%) | 0.12 |
| Radiotherapy, n(%) | 141 (38.7%) | 112 (53.8%) | <0.001 | 158 (43.4%) | 105 (50.5%) | 0.102 |
| Systemic therapy. n(%) | 181 (49.7%) | 132 (63.5%) | 0.001 | 183 (50.3%) | 131 (63.0%) | 0.003 |
| Treatment initial | | | | | | |
| atypical/wedge resection, n(%) | 29 (8.0%) | 33 (15.9%) | 0.004 | 29 (8.0%) | 13 (6.3%) | 0.449 |
| Anatomic resection, n(%) | 89 (24.5%) | 55 (26.4%) | 0.589 | 80 (22.0%) | 54 (26.0%) | 0.279 |
| Radiotherapy, n(%) | 63 (17.3%) | 45 (21.6%) | 0.203 | 71 (19.5%) | 41 (19.7%) | 0.952 |
| Systemic therapy, n(%) | 118 (32.4%) | 94 (45.2%) | 0.002 | 139 (38.2%) | 101 (48.6%) | 0.016 |

Tables S1: Comparison between PRE and POST overall and initial treatment for bronchiectasis and controls. PRE bronchiectasis: bronchiectasis diagnosed at the time of lung cancer diagnosis; PRE control: no bronchiectasis diagnosed at the time of lung cancer diagnosis; POST bronchiectasis: bronchiectasis diagnosed after lung cancer diagnosis; POST control: no bronchiectasis diagnosed after lung cancer diagnosis.
